# Enriched sera protein profiling for detection of non-small cell lung cancer biomarkers

**DOI:** 10.1186/1477-5956-9-55

**Published:** 2011-09-19

**Authors:** Emanuela Monari, Christian Casali, Aurora Cuoghi, Jessica Nesci, Elisa Bellei, Stefania Bergamini, Luca I Fantoni, Pamela Natali, Uliano Morandi, Aldo Tomasi

**Affiliations:** 1 Department of Laboratory Medicine, Medical Faculty, University of Modena and Reggio Emilia, Via del Pozzo 71, 41100, Modena, Italy; 2 Division of Thoracic Surgery, Department of General Surgery and Surgical Specialties, University of Modena and Reggio Emilia, Via del Pozzo 71, 41100, Modena, Italy

## Abstract

**Background:**

Non Small Cell Lung Cancer (NSCLC) is the major cause of cancer related-death. Many patients receive diagnosis at advanced stage leading to a poor prognosis. At present, no satisfactory screening tests are available in clinical practice and the discovery and validation of new biomarkers is mandatory. Surface Enhanced Laser Desorption/Ionization Time-of-Flight Mass Spectrometry (SELDI-ToF-MS) is a recent high-throughput technique used to detect new tumour markers. In this study we performed SELDI-ToF-MS analysis on serum samples treated with the ProteoMiner™ kit, a combinatorial library of hexapeptide ligands coupled to beads, to reduce the wide dynamic range of protein concentration in the sample. Serum from 44 NSCLC patients and 19 healthy controls were analyzed with IMAC30-Cu and H50 ProteinChip Arrays.

**Results:**

Comparing SELDI-ToF-MS protein profiles of NSCLC patients and healthy controls, 28 protein peaks were found significantly different (p < 0.05), and were used as predictors to build decision classification trees. This statistical analysis selected 10 protein peaks in the low-mass range (2-24 kDa) and 6 in the high-mass range (40-80 kDa). The classification models for the low-mass range had a sensitivity and specificity of 70.45% (31/44) and 68.42% (13/19) for IMAC30-Cu, and 72.73% (32/44) and 73.68% (14/19) for H50 ProteinChip Arrays.

**Conclusions:**

These preliminary results suggest that SELDI-ToF-MS protein profiling of serum samples pretreated with ProteoMiner™ can improve the discovery of protein peaks differentially expressed between NSCLC patients and healthy subjects, useful to build classification algorithms with high sensitivity and specificity. However, identification of the significantly different protein peaks needs further study in order to provide a better understanding of the biological nature of these potential biomarkers and their role in the underlying disease process.

## Background

Lung cancer is the leading cause of cancer-related deaths worldwide [1]. More than 80% of lung cancer patients are affected by non small cell lung cancer (NSCLC), while the remaining 20% by small cell lung cancer (SCLC). Most of lung cancer cases are diagnosed in advanced stages, and only one third of patients with new diagnosis can undergo surgical treatment that, at present, is the therapeutic option associated to the best survival rate (5-ys 70% for Stage I after surgical resection). Many efforts have been made in the last decade to improve the percentage of diagnosis at early stage, as both the chest radiography and the High Resolution Computed Tomography (HRCT) have proved to be inadequate screening tests [2]. Thus, is necessary to discover reliable biomarkers for an early and accurate diagnosis of the tumor condition.

Biomarker discovery in biological fluids, such as serum, plasma and urine, is one of the most challenging aspects of proteomic research. Most investigators believe that, due to heterogeneity of cancer diseases (histological grade, tumor stage, patient age, sex and genetic background), a set of biomarkers, instead of a single cancer-specific marker, might be more useful in clinical practice [3,4].

Surface-Enhanced Laser Desorption/Ionization Time-of-Flight Mass Spectrometry (SELDI-ToF-MS) is a relatively new proteomic technology regarded as one of the most powerful tools for differential expression profiling. This is a high through-put technique that allows obtaining protein profiles from several complex biological samples, with minimal requirements for purification and separation, in a rapid and efficient way. Small amount of sample (such as body fluids or tissue cell lysate) is directly applied on biochips, available with different chromatographic surfaces (ProteinChip Arrays). Selectively retained proteins are then directly analyzed by laser desorption and ionization. The result is a mass spectrum comprised of the mass to charge (m/z) ratio and intensities of the bound peptide/protein [5]. Afterward, the statistical analysis of the obtained protein profiles permits to reveal any protein changes, with high sensitivity and specificity. One of the key feature of SELDI-ToF-MS analysis is its ability to detect a large number of low-molecular weight proteins ( < 20 kDa), thus overcoming one of the major limitation of the two-dimensional gel electrophoresis [6,7]. Furthermore, other mass spectrometry techniques (such as MALDI or ESI) may require pre-digestion of whole proteins with enzymes (for example trypsin) in order to generate peptides small enough to be analyzed. There are however drawbacks of SELDI-ToF-MS techniques such as competitive binding of high abundance non-informative proteins to the ProteinChip surfaces, and the need of a second step to identify the protein peaks of interest.

In the last years, this approach has been used to discover potential biomarkers for the diagnosis of some types of cancer, such as ovarian [8,9], breast [10,11], colorectal [12], and prostate cancer [13,14], as well as for many other diseases [4,15-18]. Recently, SELDI-ToF-MS profiling has been applied in a few studies aimed to identify lung cancer biomarkers analyzing tissue [19-22] and serum samples [23-28]. Unfortunately, although the obtained results are quite promising, so far none of the discovered biomarker has already been validated for the clinical use. It is well known that the discovery of protein biomarkers from serum samples is complicated due to its wide dynamic range (over 10 orders of magnitude). In addition, the few high-abundant blood species constitute 95% of the total protein content, representing, at the same time, less than 0.1% of the total proteins [3,29], making very difficult the detection of the low-abundant components. The ProteoMiner™ technology is a novel approach, consisting of a combinatorial library of beads-coupled hexapeptide ligands, that assures the capture of all protein species present in a proteome enhancing the concentration of the most dilute ones [30-32]. The beads work on the principle of solid-phase affinity adsorption; each specie has theoretically the same probability to bind to its high affinity ligand. The most abundant proteins quickly saturated their binding sites, while the low and medium abundance proteins are concentrated on their specific ligands. At the end, only retained proteins are eluted and collected, while the excess proteins are washed away.

No depletion of any species is contemplated by this methodology, but a reduction of the relative concentration of the abundant components and a strong decrease of the sample dynamic range.

In this study, before investigating the proteomic profile of patients with NSCLC in comparison with healthy subjects, we treated serum samples with the ProteoMiner™ kit. Our goal was to verify, by SELDI-ToF-MS analysis, the presence of specific protein patterns in enriched serum samples, able to discriminate NSCLC patients from healthy subjects.

## Methods

### Study population and clinical specimens

Whole blood samples (10 mL) were collected immediately before surgery in a test tube and allowed to clot at room temperature for 1 h. After centrifugation at 2000 × g for 10 min at 4°C, serum was divided in aliquots and immediately stored at -80°C until use.

Serum samples from 44 NSCLC patients and 19 from healthy subjects were analyzed.

All NSCLC cases were candidate to complete surgical resection. Moreover, no patient underwent induction chemotherapy before surgery. The histological distribution was: 28 adenocarcinomas and 16 squamous carcinomas (histological diagnosis according to the World Health Organization 2004 classification of lung tumors). Patients were staged according to the new 2009 IASLC (International Association for the Study of Lung Cancer) staging system. Pathologic stage distribution was as follows: 15 stage IA, 12 stage IB, 5 stage IIA, 4 stage IIB, 7 stage IIIA, and 1 stage IIIB (multiple nodules in different lobes). The mean age was 71 years, (range 51-88 years, STD 8 years); 9 female and 35 males. Nineteen healthy volunteers were selected as controls; the mean age was 68 years; range 47-82 years, STD 10 years, 4 females and 13 males (no statistical difference was present between cases and control regarding gender and age). The main inclusion criteria for this group was the absence of pulmonary diseases proved by a recent chest X-Ray. The present study was performed according to a protocol approved by the Ethical Committee of the University Hospital of Modena, Italy. Voluntary informed consent to donate serum was obtained for all research participants.

### Protein enrichment

ProteoMiner™ Protein Enrichment kit (BioRad Laboratories Inc., Hercules, CA, USA) was utilized according to the manufacturer instructions. Briefly, 1 mL of serum was added to spin columns containing the beads and incubated at room temperature for 2 h with constant end-to-end rotation. After columns wash, the proteins bound to the beads were eluted with the appropriate buffer, divided in aliquots and stored at -20°C until use. In order to obtain a quality control (QC) sample as similar as possible to the analyzed samples, 20 μL of each sample treated with the ProteoMiner™ kit were pooled and used for all the experiments. Protein concentration of each sample was assessed using an assay based on the Bradford method [33].

### SELDI-ToF-MS protein profiling

Enriched serum samples were analyzed with SELDI-ToF-MS, with the purpose to investigate the protein profile in both the low (2-30 kDa) and the high (30-100 kDa) molecular weights (MW). In a preliminary study, in order to set up the experimental conditions, pooled serum samples were loaded onto three different types of ProteinChip Arrays (Bio-Rad Laboratories Inc., Hercules, CA, USA): H50 (that binds proteins through reverse-phase or hydrophobic interactions), CM10 (negatively charged surface that acts as a weak cation-exchanger) and IMAC30-Cu (Immobilized Metal Affinity Capture surface pre-activated with copper). The CM10 array gave the lower number of peaks detected (~ less than 10%) and the lower total signal intensity (~ less than 50%) compared to H50 and IMAC30-Cu, so only these two arrays were used in the main study.

In order to minimize any bias sources, each sample was randomly loaded in duplicate in a 96 well bioprocessor. Moreover, to assess the reproducibility, a QC sample was included in each ProteinChip array and all steps were automated using a robotic instrument for liquid handling (Biomek 3000 Laboratory Automation Workstation, Beckman Coulter, Fullerton, CA, USA).

Ten microliters of diluted serum sample (3 μg/μL final concentration) were mixed with 90 μL of binding buffer and loaded onto pre-equilibrated ProteinChip Array spot surfaces. After 45 min incubation at room temperature with constant horizontal shaking, the unbound proteins were removed by three washing steps using 200 μL of the same binding buffer. Finally, 1 μL of saturated sinapinic acid solution in 0.5% trifluoroacetic acid and 50% acetonitrile (Sigma-Aldrich, St.Louis, MO, USA) was applied to each spot twice, allowing the surface to dry between each application.

### Data acquisition

The ProteinChip Arrays were analyzed with a SELDI-ToF-MS reader (Series 4000, Bio-Rad Laboratories Inc., Hercules, CA, USA), by protocols optimized for low and high MW ranges. Protein mass spectra were generated using an average of 901 laser shot for each protocol. The "All-in-one protein standard II" (Bio-Rad Laboratories Inc., Hercules, CA, USA) was used to obtain protein standard spectra for mass accuracy calibration.

### Statistical analysis

Statistical analysis was performed using the ProteinChip Data Manager 3.0 software (Bio-Rad Laboratories Inc., Hercules, CA, USA). The spectra were mass calibrated, baseline subtracted, mass aligned and finally normalized by total ion current in both the MW ranges of interest. All poor quality spectra were excluded from the statistical analysis. Supervised clustering was performed using the following settings: 5 times signal-to-noise (S/N) ratio and 20% min peak threshold in the first pass for peaks identification, and 2 times S/N ratio on the second pass for cluster completion.

After clusters identification, to test the null hypothesis that the medians of peak intensities of the two groups were equal, Mann Whitney U test was performed. A p-value less than 0.05 was accepted as statistically significant.

To test the overall quality of the assay, QC sample spectra replicates were used to calculate pooled CV%. This was obtained, for each protocol, using the intensity CV % of 20 representative cluster peaks, regularly distributed for mass ranges and peak intensities, including all the statistical significant peaks. The pooled CV % means were 23.4% and 24.5% in the low-mass range, and 22.2% and 24.1% in the high- mass range, for IMAC30-Cu and H50, respectively.

### Decision tree classification

Decision tree classification was performed using Biomarker Pattern Software 5.0 (Ciphergen, USA) based on CART (Classification And Regression Trees) as described by Breiman et al. [34]. Classification tree split up a data set into nodes using one rule at a time. For each node the decision is made by the presence or absence or the intensity level of one peak until a terminal node is reached or further splitting has no gains. Classification was performed using as target the group (class), the Gini method, and 10-fold cross-validation, and as predictors all the peaks with a statistical significance between NSCLC and controls (p-value < 0.05). Peaks selected by this process are the ones present in the lowest cost trees. The 10-fold cross- validation test divided the data set into approximately 10 equal subsets. The tree-growing process is repeated from scratch 10 times and in each cross-validation replication nine subsets of the data are used as learning data and one subset is used as a test sample. At the end of process, the error counts from each of the 10 test samples are summed to obtain the overall error count for each tree in the full-sample tree sequence.

## Results

A total of 63 enriched serum samples were analyzed by SELDI-ToF-MS with both IMAC30-Cu and H50 ProteinChip Arrays. For each reading protocol, the MS spectra were processed as described above. A total of 106 cluster peaks (88 in the low- and 18 in the high-MW) for IMAC30-Cu, and 95 peaks (73 in the low- and 22 in the high-MW) for H50 were detected. Non-parametric Mann-Witney U test was carried out to verify the presence of peaks with statistically significant relative intensities between NSCLC patients and control subjects. Using IMAC30-Cu, 9 significant peaks (6 in the low-MW range and 3 in the high-MW range) were found comparing NSCLC patients to controls. With H50 ProteinChip Array, 19 significant peaks (14 in the low-MW and 5 in the high-MW) were obtained. These differentially expressed peaks (Additional file 1, Table S1) were used to construct decision trees classification algorithms, in the attempt to identify potential serum biomarkers for NSCLC.

**Figure 1:**
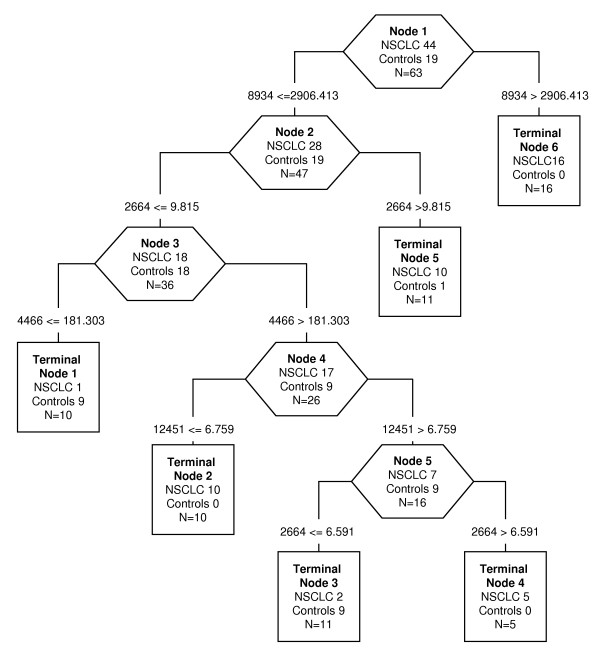
**Decision tree classification diagram of low-MW serum protein peaks from NSCLC patients and controls, applying the IMAC30-Cu conditions**. The numbers in the root (top), descendant nodes (exagons) and terminal nodes (rectangles), represent the classes (NSCLC and controls, N = sum of NSCLC and controls subjects). The numbers below the root and the descendant nodes indicate the values of mass peak and its intensity, respectively.

**Figure 2:**
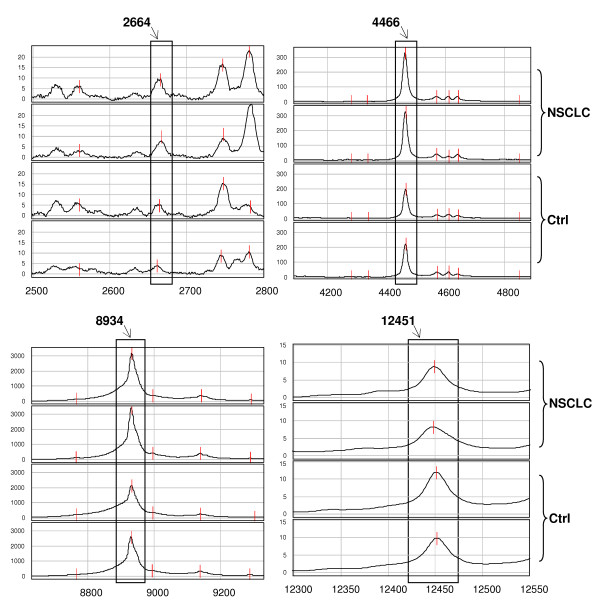
**Serum protein profile of low-MW predictor peaks (IMAC30-Cu)**. Representative spectra (in duplicate) obtained by SELDI-ToF-MS analysis concerning the 4 statistically significant peaks detected with IMAC30-Cu and used as predictors to build the decision classification tree shown in Figure 1. The peaks of interest are highlighted in rectangles and their m/z values are reported above. *(Ctrl = control subjects)*.

For IMAC30-Cu, 6 statistically different protein peaks in the low-MW range were chosen as predictor to build up the decision classification tree shown in Figure 1. The classification algorithm used 4 protein peaks and 6 terminal node were determined with a relative cost of 0.61. The accuracy of this algorithm was 93.65% (59/63), which correctly classified 41 of 44 NSCLC (93.18%) and 18 of 19 healthy controls (94.74%). The accuracy after 10-fold cross-validation was 69.84%, with sensitivity of 70.45% (31/44) and specificity of 68.42% (13/19). In Table 1 are listed the 4 protein peaks: 3 were up-regulated (2664, 4466, 8934 m/z), and 1 was down-regulated (12451 m/z) in NSCLC patients. Figure 2 shows representative spectra of these protein peaks in NSCLC patients and controls.

**Table 1:** Comparison of low-MW predictor protein peaks intensities (IMAC30-Cu) between NSCLC patients and controls

m/z	NSCLC (mean ± SD)			Controls (mean ± SD)			p-value
2664	8.672	±	4.215	6.142	±	2.948	0.020
4466	234.807	±	56.203	195.299	±	63.867	0.046
8934	2503.941	±	516.768	2303.279	±	247.386	0.022
12451	4.182	±	3.304	6.471	±	4.294	0.036

**Figure 3:**
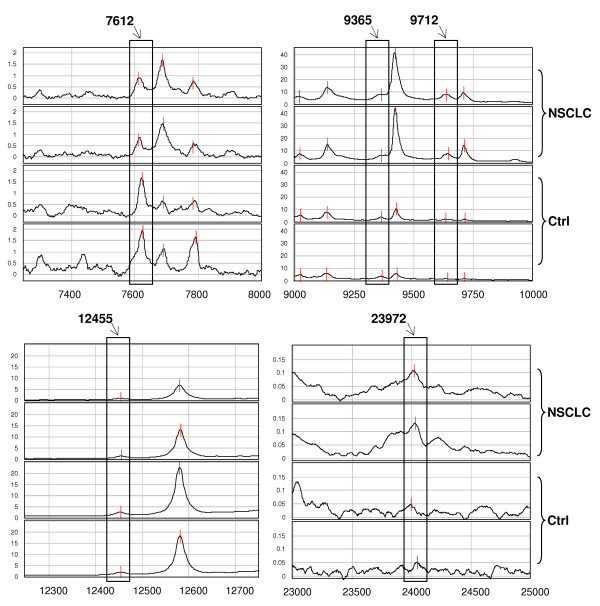
**Serum protein profile of low-MW predictor peaks (H50)**. Representative spectra (in duplicate) obtained by SELDI-ToF-MS analysis concerning the 5 statistically significant peaks detected with H50 ProteinChip Array and used as predictors to build the decision classification tree shown in Figure 4. The peaks of interest are highlighted in rectangles and their m/z values are reported above. *(Ctrl = control subjects)*.

**Figure 4:**
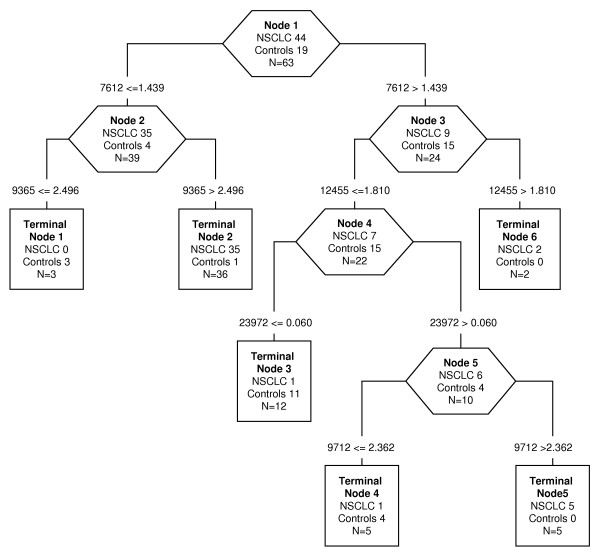
**Decision tree classification diagram of low-MW protein peaks from NSCLC patients and controls, using H50 conditions**. The numbers in the root (top), descendant nodes (exagons) and terminal nodes (rectangles), represent the classes (NSCLC patients and controls, N = sum of NSCLC and controls). The numbers below the root and descendant nodes indicate the values of mass peak and its intensity, respectively.

The same analysis was carried out with H50 ProteinChip Array, using as predictors 14 significant peaks found in the low-MW range; the classification algorithm selected 5 protein peaks, reported in Table 2. Three of these peaks were up-regulated (9365, 9712, and 23972 m/z) and 2 were down-regulated (7612 and 12455 m/z) in NSCLC patients compared with controls. Representative spectra are shown in Figure 3. The classification decision tree generated 6 terminal nodes with a relative cost of 0.536 (Figure 4). The decision algorithm correctly classified 42 of 44 NSCLC patients (95.45%) and 18 of 19 controls (94.74%), with an accuracy of 95.23% (60/63). After crossing validation, the accuracy decreased to 73.01% (46/63) with sensitivity of 72.73% (32/44) and specificity of 73.68% (14/19).

**Table 2:** Comparison of low-MW predictor protein peaks intensities (H50) between NSCLC patients and controls

m/z	NSCLC (mean ± SD)			Controls (mean ± SD)			p-value
7612	1.051	±	0.681	1.847	±	0.836	0.001
9365	4.269	±	1.403	3.353	±	0.871	0.019
9712	3.764	±	2.797	1.919	±	0.518	0.002
12455	0.702	±	0.674	0.886	±	0.483	0.019
23972	0.064	±	0.025	0.052	±	0.033	0.030

Decision classification trees were also obtained using as predictors the statistically significant peaks detected in the high-mass range (30-100 kDa) for IMAC30-Cu (Additional File 2, Figure S1) and H50 (Additional File 3, Figure S2). Two peaks were selected by this process for IMAC30-Cu and 3 peaks for H50, respectively (Table 3). In this case, the relative cost and the misclassification rate were higher than those obtained in the low-MW. Actually, after 10-fold-cross-validation, 31 of 44 NSCLC patients (70.45%) and 6 of 19 control subjects (68.42%) using IMAC30-Cu, and 21/44 NSCLC (47.73%) and 9 of 19 (52.63%) controls with H50 ProteinChip Arrays, were correctly classified.

**Table 3:** Comparison of high-MW range predictor peak intensities between NSCLC patients and controls for IMAC30-Cu and H50

m/z	NSCLC (mean ± SD)			Controls (mean ± SD)			p-value
***IMAC30-Cu***							
45973	1.514	±	0.448	1.097	±	0.331	0.001
80313	0.134	±	0.073	0.172	±	0.094	0.050

***H50***							
34527	2.346	±	0.569	2.039	±	0.497	0.036
51996	0.327	±	0.326	0.255	±	0.338	0.026
73503	0.128	±	0.010	0.066	±	0.031	0.025

Finally, all the 28 statistically significant peaks, identified in all experimental condition, were used as predictors to build an unique decision classification tree (Additional File 4, Figure S3). This algorithm used 5 protein peaks: 3 already selected in other decision algorithms (7612, 8934 and 12455 m/z) and 2 new peaks (12588 and 44689 m/z) (Additional File 5, Table S2).

The decision algorithm correctly classified 40 of 44 NSCLC patients (90.91%) and 18 of 19 controls (94.74%), with an accuracy of 92.06% (58/63). After crossing validation, the accuracy decreased to 61.90% (39/63) with sensitivity of 65.91% (29/44) and specificity of 52.63% (10/19).

## Discussion

The incidence of lung cancer is constantly increasing, with an high mortality rate due to delay in diagnosis. For this reason, early NSCLC biomarkers could be crucial for the diagnosis, prognosis and follow-up. Many studies have been made in the past years in the attempt to discover reliable biomarkers, but, to date, their poor organ and tumor specificity limits their use to prognosis and therapy monitoring [35].

In order to discover novel protein biomarkers, a number of different technologies are used. Among these techniques, SELDI has the advantage to allow direct protein profiles of biological fluids (such as serum or urine) in a rapid and reproducible way. This generate an huge amount of data that can be directly analyzed with the bioinformatics tools coupled with the system.

Although this technology was successfully applied for the discovery of candidate biomarker in different tumor types, currently, in literature, only few SELDI-ToF-MS studies on lung cancer are reported, especially performed on crude serum samples, without any preliminary pre-fractionation or depletion treatment. For example, Han et al. [26] analyzed, on H4 ProteinChip Array, untreated serum from patients with SCLC, NSCLC, pneumonia and from healthy individuals, defining 3 different protein patterns able to discriminate SCLC from controls and the different diseases with each other. Some authors used CM10 ProteinChip Array to compare crude serum of lung cancer patients and healthy controls. Although the same ProteinChip type was used, they discovered different protein peaks, proposed as candidate biomarkers. Dai et al, in fact, identified a protein peak at 11.6 kDa (serum Amyloid A protein), able to discriminate lung cancers from controls with a sensitivity of 84% and specificity of 80% [25]. Yang et al. [23] detected 5 protein peaks at m/z 11493, 6429, 8345, 5335 and 2538 that were chosen to build a classification algorithm. It permitted to discriminate stages I and II of NSCLC with a sensitivity of 91.4% and 79.1%, respectively. More recently, Yang et al. [28] discovered and validate 3 candidate biomarkers in NSCLC: one down-regulated, identified as apolipoprotein C-I (6628 Da), and 2 up-regulated, haptoglobin alpha-1 chain (9191 Da) and S100A4 (11412 Da). Moreover, other ProteinChip Arrays, such as IMAC-30, were used to perform SELDI-ToF analysis on crude serum samples from lung cancer patients [27].

The only work on pretreated serum samples is by Au et al. [24]: they used the Equalizer beads, the developing combinatorial library ligands technology that was then commercialized with the trade name ProteoMiner. They treated serum from never-smoked lung cancer patients and normal control subjects using IMAC30 and Q10 ProteinChip Array. Comparing the serum proteomic profiles of patients with controls, they found several statistically significant protein peaks, mostly in the high mass-range ( > 50 kDa).

Unlike the majority of the previous studies reported in literature, we conducted a SELDI-ToF-MS analysis on serum samples treated with commercially available ProteoMiner™kit. This technique has the main advantage to reduce the serum high dynamic range by lowering the concentration of most abundant protein species and simultaneously concentrating the less abundant ones. Other different depletion methods, based on dye-ligands or specific antibodies, are currently available but they could produce some drawbacks, such as co-depletion [36]. It is widely demonstrated that the ProteoMiner™ technique is able to increase the recovery yield of protein species detected with two-dimensional gel electrophoresis and SELDI-ToF-MS analysis, preserving their proportionality. This permits to reveal low and medium abundance proteins in serum and plasma samples, as needed for biomarker discovery [37-39].

In the present work we treated with this new approach serum samples from 44 NCSLC and 19 healthy controls, obtaining 106 cluster of protein peaks for IMAC30-Cu and 95 for H50 ProteinChip Arrays, respectively. The comparison of the clusters between the two group identified 28 cluster peaks (20 in the low and 8 in the high mass range) statistically different (p < 0.05) and they were used as predictors to build decision classification algorithms (4 classifications built considering ProteinChip type and MW separately, and 1 built considering all conditions). These analyses selected 4 peaks in the low- and 2 peaks in the high-MW for IMAC30-Cu, and 5 in the low- and 3 in the high-MW for H50. The classification models for the low-mass range after 10-fold cross-validation had a sensitivity of 70.45% (31/44) and a specificity of 68.42% (13/19) for IMAC30-Cu, and 72.73% (32/44) sensitivity and 73.68% (14/19) specificity for H50 ProteinChip Array. The classification algorithm built with all cluster identified in all experimental conditions allowed to single out two more peaks, even if this algorithm had a lower classification power, 65.91% (29/44) sensitivity and 52,63% (10/19) specificity.

When compared with other studies present in literature, the use of ProteoMiner™ kit permits to increase the number of significant peaks able to discriminate NSCLC patients from healthy controls. In fact, although the limited number of enrolled subjects and considering the relatively heterogeneity of cases regarding disease stage, a total of 16 interesting protein peaks were discovered, mostly in the low mass range. It is also important to note that all cases enrolled came from a surgical series and all patients with a clearly metastatic disease (stage IV) were excluded. This point addresses the possibility to discover potential candidate biomarkers to differentiate patients amenable of a surgical treatment at diagnosis, that is the therapeutic option associated to the best survival rate.

## Conclusions

In summary, in our study we used the ProteoMiner™ kit prior to SELDI-ToF-MS analysis to reduce the complexity of NSCLC and controls serum samples. Statistical analysis of differentially expressed protein peaks has permitted to build algorithms, that could discriminate between NSCLC patients and control subjects, with high rate of sensitivity and specificity. Our results show that the SELDI-ToF-MS technology coupled with the ProteoMiner™ strategy is able to identify a set of protein peaks as candidate biomarkers, in a rapid and high-throughput mode. These protein peaks could be useful to select, among patients at risk to develop lung cancer (such as heavy smokers > 40 years), those that require an aggressive radiological follow up, in order to discover the neoplastic condition at an early stage. However, further studies, increasing the number of patients and controls, are needed to confirm these results and especially to identify and subsequently validate the discovered protein peaks.

## List of abbreviations

NSCLC: Non Small Cell Lung Cancer; SCLC: small cell lung cancer; SELDI-ToF-MS: Surface Enhanced Laser Desorption/Ionization Time-of-Flight mass spectrometry.

## Competing interests

The authors declare that they have no competing interests.

## Authors' contributions

EM performed proteomics experiments and wrote the manuscript, CC, JN and PN selected clinical cases and collected serum samples, AC and EB performed proteomics experiments, SB and LIF carried out samples preparation for mass spectrometry analysis, UM and AT supervised the work, provided useful suggestions to improve performance, and revised the manuscript.

All authors have seen a draft copy of the manuscript and agree with its publication.

## Supplementary Material

Additional file 1 **Table S1 -Comparison of mass peak intensities between NSCLC and Controls**. Click here for file

Additional file 2 **Figure S1- Diagram of the decision tree classification of serum protein peaks between NSCLC and Controls in IMAC30-Cu high-mass range condition**. Click here for file

Additional file 3 **Figure S2 - Diagram of the decision tree classification of serum protein peaks between NSCLC and Controls in H50 high-mass range condition**. Click here for file

Additional file 4 **Figure S3 - Diagram of the decision tree classification for all significant protein peaks between NSCLC and Controls**. Click here for file

Additional file 5 **Table S2 -Comparison of mass peak intensities between NSCLC and Controls**. Click here for file
