# Supplementary material for: Enriched sera protein profiling for detection of non-small cell lung cancer biomarkers
Source: Proteome Sci. 2011 Sep 19;9:55. doi: 10.1186/1477-5956-9-55 (PMC3184051; doi:10.1186/1477-5956-9-55)
Supplement: Additional file 1 — Table S1 -Comparison of mass peak intensities between NSCLC and Controls. [file 1477-5956-9-55-S1.PDF]

**Table S1**

Comparison of mass peak intensities between patients with NSCLC and Controls individuals for IMAC30-Cu and H50 protein chips

| m/z | Patients with NSCLC (mean ± SD) | | | Controls individuals (mean ± SD) | | | p-value |
|---|---|---|---|---|---|---|---|
| ***IMAC30-Cu*** | | | | | | | |
| 2664 | 8.672 | ± | 4.215 | 6.142 | ± | 2.948 | 0.020 |
| 2782 | 32.122 | ± | 23.178 | 22.699 | ± | 26.099 | 0.026 |
| 4466 | 234.807 | ± | 56.203 | 195.299 | ± | 63.867 | 0.046 |
| 8934 | 2503.941 | ± | 516.768 | 2303.279 | ± | 247.386 | 0.022 |
| 9141 | 343.413 | ± | 55.394 | 309.133 | ± | 42.918 | 0.015 |
| 12451 | 4.182 | ± | 3.304 | 6.471 | ± | 4.294 | 0.036 |
| 44689 | 1.378 | ± | 0.385 | 1.183 | ± | 0.464 | 0.008 |
| 45973 | 1.514 | ± | 0.448 | 1.097 | ± | 0.331 | 0.001 |
| 80313 | 0.134 | ± | 0.073 | 0.172 | ± | 0.094 | 0.050 |
| ***H50*** | | | | | | | |
| 4402 | 2.868 | ± | 1.171 | 2.227 | ± | 0.650 | 0.046 |
| 6287 | 3.463 | ± | 1.544 | 4.381 | ± | 1.785 | 0.035 |
| 6438 | 18.215 | ± | 8.390 | 23.464 | ± | 8.800 | 0.015 |
| 6535 | 1.931 | ± | 0.584 | 2.398 | ± | 0.782 | 0.021 |
| 7612 | 1.051 | ± | 0.681 | 1.847 | ± | 0.836 | 0.001 |
| 8935 | 25.291 | ± | 17.581 | 14.847 | ± | 5.183 | 0.040 |
| 9365 | 4.269 | ± | 1.403 | 3.353 | ± | 0.871 | 0.019 |
| 9712 | 3.764 | ± | 2.797 | 1.919 | ± | 0.518 | 0.002 |
| 12455 | 0.702 | ± | 0.674 | 0.886 | ± | 0.483 | 0.019 |
| 12588 | 4.843 | ± | 3.664 | 7.100 | ± | 3.861 | 0.013 |
| 12887 | 13.053 | ± | 9.804 | 22.807 | ± | 18.048 | 0.011 |
| 13092 | 2.337 | ± | 1.399 | 3.649 | ± | 2.238 | 0.011 |
| 23972 | 0.064 | ± | 0.025 | 0.052 | ± | 0.033 | 0.030 |
| 26105 | 0.202 | ± | 0.112 | 0.127 | ± | 0.027 | 0.001 |
| 34527 | 2.346 | ± | 0.569 | 2.039 | ± | 0.497 | 0.036 |
| 45430 | 1.776 | ± | 0.428 | 1.528 | ± | 0.27527 | 0.049 |
| 51996 | 0.327 | ± | 0.326 | 0.255 | ± | 0.338 | 0.026 |
| 54263 | 0.372 | ± | 0.222 | 0.245 | ± | 0.098 | 0.037 |
| 73503 | 0.128 | ± | 0.010 | 0.066 | ± | 0.031 | 0.025 |
