# Supplementary material for: Enriched sera protein profiling for detection of non-small cell lung cancer biomarkers
Source: Proteome Sci. 2011 Sep 19;9:55. doi: 10.1186/1477-5956-9-55 (PMC3184051; doi:10.1186/1477-5956-9-55)

Fig. S2

Diagram of the decision tree classification in the H50 high-mass range condition. The numbers in the root (top), descendant nodes (exagons) and terminal nodes (rectangles) represent the classes (NSCLC= Non Small Cell Lung Cancer, Controls, N= sum of NSCLC and Controls). The numbers below the root and descendant nodes are the mass values followed by the peak intensity decision value.

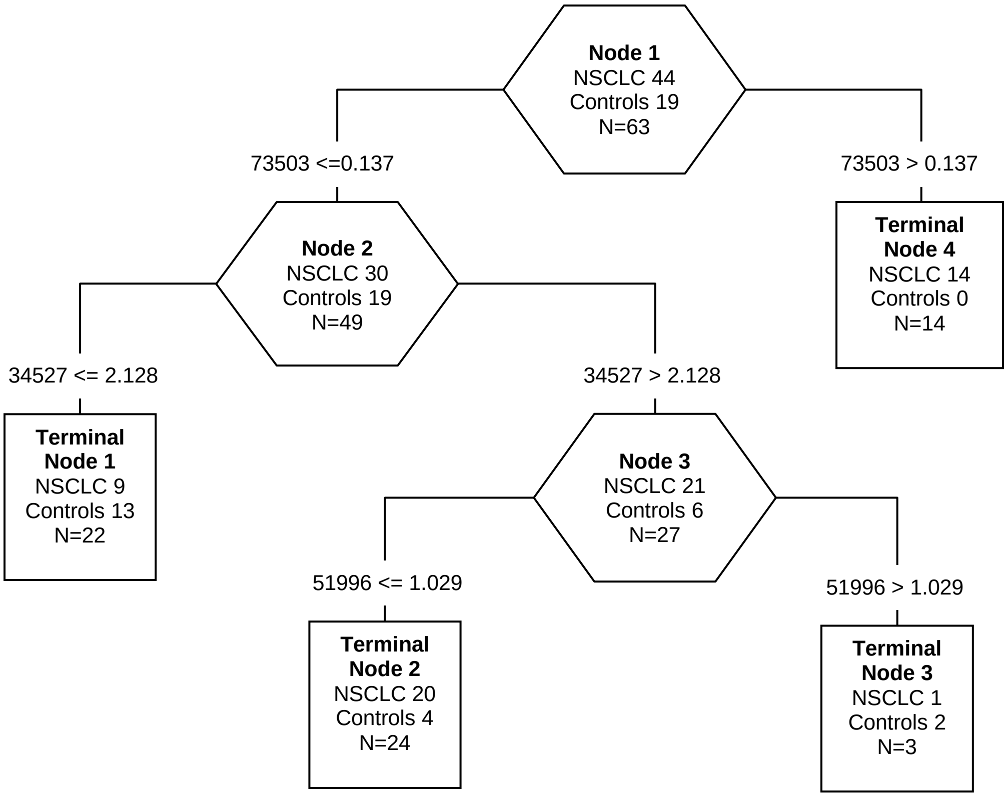

Supplement: Additional file 3 — Figure S2 - Diagram of the decision tree classification of serum protein peaks between NSCLC and Controls in H50 high-mass range condition. [file 1477-5956-9-55-S3.PDF]
