# Supplementary material for: Enriched sera protein profiling for detection of non-small cell lung cancer biomarkers
Source: Proteome Sci. 2011 Sep 19;9:55. doi: 10.1186/1477-5956-9-55 (PMC3184051; doi:10.1186/1477-5956-9-55)
Supplement: Additional file 5 — Table S2 -Comparison of mass peak intensities between NSCLC and Controls. [file 1477-5956-9-55-S5.PDF]

Table S2

Comparison of mass peak intensities between patients with NSCLC and Controls for all cluster (2-100 kDa molecular range).

m/z	NSCLC (mean ± SD)	Controls (mean ± SD)	p-value
7612	1.051 ± 0.681	1.847 ± 0.836	0.001
8934	2503.941 ± 516.768	2303.279 ± 247.386	0.022
12455	0.702 ± 0.674	0.886 ± 0.483	0.019
12588	4.843 ± 3.664	7.100 ± 3.861	0.013
44689	1.378 ± 0.385	1.183 ± 0.464	0.008
